# Topical Administration of Sitagliptin Prevents Retinal Neurodegeneration in a Model of Glaucoma Induced by Dexamethasone

**DOI:** 10.3390/ijms27010048

**Published:** 2025-12-20

**Authors:** Patricia Bogdanov, Anna Duarri, David Sabater, María José Canz, Helena Isla-Magrané, Hugo Ramos, Anna Deàs-Just, Rafael Simó, Cristina Hernández

**Affiliations:** 1 Diabetes and Metabolism Research Unit, Vall d’Hebron Research Institute, Universitat Autònoma de Barcelona, 08035 Barcelona, Spain; david.sabater@vhir.org (D.S.); maria.canz@vhir.org (M.J.C.); hugo.ramos@vhir.org (H.R.); anna.deas@vhir.org (A.D.-J.); cristina.hernandez@vhir.org (C.H.); 2 Centro de Investigación Biomédica en Red de Diabetes y Enfermedades Metabólicas Asociadas (CIBERDEM), Instituto de Salud Carlos III (ISCIII), 28029 Madrid, Spain; 3 Ophthalmology Research Group, Vall d’Hebron Institut de Recerca (VHIR), 08035 Barcelona, Spain; anna.duarri@vhir.org (A.D.); helena.isla@gmail.com (H.I.-M.)

**Keywords:** primary open-angle glaucoma, dexamethasone-induced experimental model, dipeptidyl peptidase 4 inhibitors, sitagliptin, eye drops

## Abstract

Glaucoma is a neurodegenerative disease characterized by progressive degeneration of optic nerve axons and loss of retinal ganglion cells (RGCs). Although elevated intraocular pressure (IOP) is a major risk factor, many patients develop glaucoma with normal IOP, highlighting the need for neuroprotective therapies. Sitagliptin, a dipeptidyl peptidase-4 inhibitor, has shown beneficial effects in diabetes-induced retinal neurodegeneration. This study aimed to evaluate whether sitagliptin eye drops, previously effective in diabetes-induced retinal neurodegeneration, could prevent corticosteroid-induced glaucoma. Glaucoma was induced in mice by periocular injection of dexamethasone (DEX) once weekly for five weeks. Sitagliptin or vehicle eye drops were administered from day 14 to 35. Untreated mice served as controls. DEX treatment caused significant loss of RGC bodies and optic nerve axons compared to controls, which was prevented by sitagliptin eye drops (*p* < 0.001), without affecting IOP. Sitagliptin also inhibited DEX-induced activation of macroglia and microglia and prevented oligodendrocyte loss. Furthermore, it suppressed overexpression of galectin-3 and gamma-synuclein in the optic nerve head (ONH) (*p* < 0.001), key mediators of inflammation and apoptosis. Sitagliptin eye drops exert a potent neuroprotective effect against corticosteroid-induced glaucoma, supporting their potential as a novel therapeutic strategy for glaucoma.

## 1. Introduction

Glaucoma, a group of diseases characterized by progressive optic neuropathy, is one of the leading causes of severe visual impairment and blindness worldwide. The global prevalence of glaucoma for the population aged 40–80 years is 3.54%, and it is estimated that it affects around 111.8 million people worldwide by 2040 [1]. This neurodegenerative disease causes the progressive degeneration of optic nerve axons and the retrograde death of retinal ganglion cells (RGCs), leading to visual impairment that may progress to blindness in 10% of cases. It should be highlighted that more than half are unaware that they are affected, as the disease often remains asymptomatic until it is severe [2]. Open-angle glaucoma comprises the majority of cases in the United States and Western Europe, of which primary open-angle glaucoma (POAG) is the most common type [3]. In addition to age and genetics, elevated IOP is a major risk factor for glaucoma, causing damage to RGCs, which serve as the ultimate output neurons of the retina and transmit visual information to the brain. The IOP results in compression of the lamina cribosa with consequent mechanical axonal damage and disruption of axonal transport that interrupts retrograde delivery of essential trophic factors to RGCs from their brainstem target [2]. However, a substantial proportion of glaucoma patients (25–50%) have a normal IOP level [2]. These patients display a similar RGC loss in the absence of high IOP and, therefore, neuroprotection rather than an IOP-lowering approach seems the most rational strategy.

In recent years, evidence has accumulated showing the brain neuroprotective effects of Glucagon-Like Peptide (GLP-1) and GLP-1 receptor agonists (GLP-1RA) in experimental studies and even in phase II clinical trials [4,5]. We have shown that GLP-1/GLP-1R are produced by the human retina and that both mRNA levels and protein content of GLP-1 were significantly lower in retinas from subjects with diabetes in comparison with control individuals [6]. In addition, we have demonstrated the neuroprotective effects of topical (eye drops) administration of GLP-1 and GLP-1 agonists in the db/db mouse model [6]. Therefore, topical GLP-1 treatment can be contemplated as a replacement treatment. Apart from administrating GLP-1, another way to increase GLP-1 within the retina is by inhibiting its degradation. GLP-1 is extremely susceptible to the catalytic activity of the enzyme dipeptidyl peptidase IV (DPP-IV), which cleaves off the two NH2-terminal amino acids. Consequently, GLP-1 rapidly degrades, showing a half-life in plasma of 1–2 min. The higher DPP-IV concentrations detected in the retinal pigment epithelium (RPE) of diabetic donors compared to non-diabetic donors could decrease the availability of GLP-1 for reaching the neuroretina [7]. In fact, drugs that reach the retina via the transscleral route, as is the case for GLP-1, are first challenged by the choroid and the RPE [8]. For all these reasons, we performed a study to demonstrate that the enhancement of the retinal content of GLP-1 by preventing its degradation could be a new strategy for treating the early stages of diabetic retinopathy (DR). In this regard, we reported in rabbits that sitagliptin topically administered reaches the retina prior to the aqueous and vitreous humors, suggesting that its absorption follows the transscleral route. Moreover, systemic absorption was minimal and below pharmacologically active concentrations [9]. In summary, we found that sitagliptin, a DPP-IV inhibitors administered in eye drops, led to a significant increase in the intraretinal content of GLP-1, thus preventing neurodegeneration and vascular leakage in db/db mice [7]. In addition, several studies suggest that DPP-IV inhibitors have neuroprotective effects unrelated to GLP-1R activation, but the underlying mechanisms are still not fully understood [9,10,11,12,13,14].

On this basis, and given that DR and glaucoma share several common pathogenic pathways in the neurodegenerative process [15,16], we examined whether the topical administration of sitagliptin could also be useful in preventing retinal neurodegeneration in a well-established mouse model of secondary glaucoma induced by periocular administration of glucocorticoids which clinically resembles primary open-angle glaucoma (POAG) [17,18].

## 2. Results

The mouse model selected for this study was non-diabetic and, therefore, the blood glucose levels were not altered (Supplementary Figure S1).

### 2.1. IOP Assessments

Glaucoma was induced in both the right and left eyes of each animal. IOP measurements in both eyes were performed weekly until the day before euthanasia. Periocular injections of DEX resulted in a significant increase in IOP (Supplementary Figure S1A). Treatment with sitagliptin had no effect on IOP. In fact, we found similar IOP in the DEX-induced glaucoma mice treated with vehicle or with sitagliptin at the end of treatment (20.8 ± 1.7 vs. 20.4 ± 0.9 mm Hg; *p* = ns). Similar results were obtained in the right and left eye.

### 2.2. Ocular Assessment

Following each periocular administration of DEX, none of the eyes exhibited signs of inflammation, such as congestion or opacity of the conjunctiva in the anterior chamber. Neither the periocular DEX treatment nor the sitagliptin eye-drops induced changes in the body weight of the animals throughout the entire study (Supplementary Figure S1F). Furthermore, no apparent vascular effects were observed in all groups when examined using fundoscopy and fundus fluorescence angiography (Supplementary Figure S1B).

### 2.3. Sitagliptin Prevented RGCs Loss

**Figure 1:**
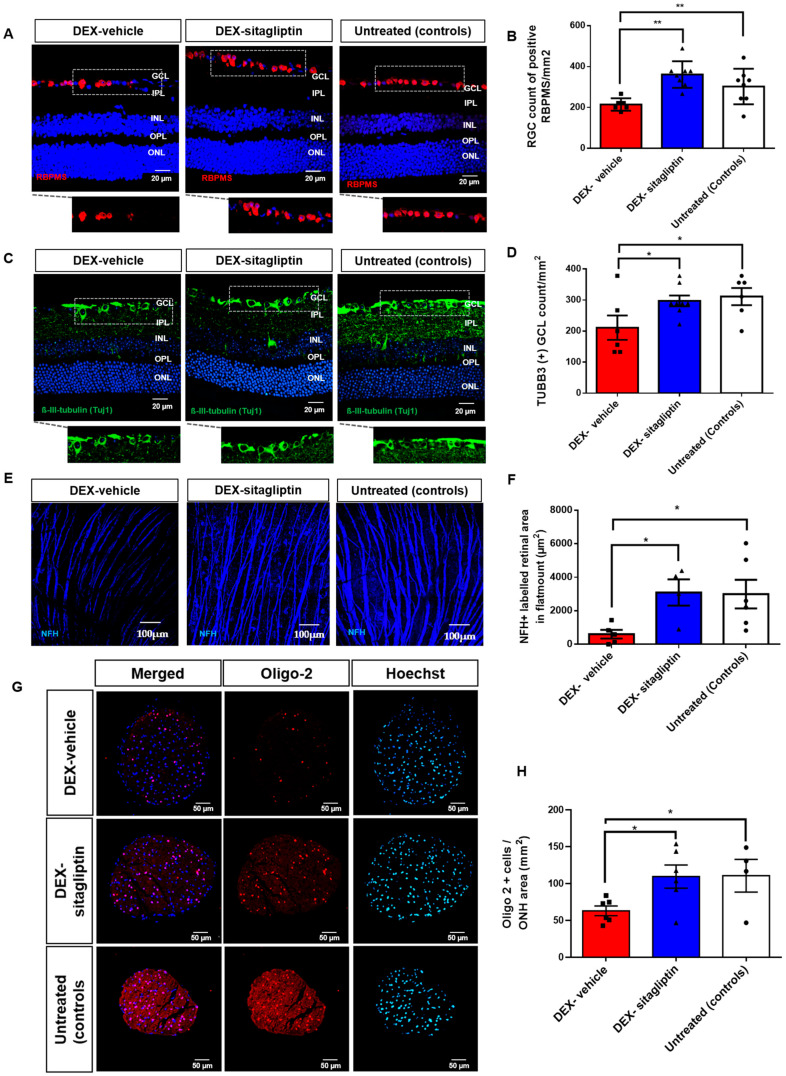
Sitagliptin prevents retinal ganglion cell (RGC) and oligodendrocyte (OL) loss in the retina and optic nerve head (ONH) of DEX-induced glaucoma mice. (**A**) Representative images of RBPMS immunoreactivity (red) in the neuroretina. Scale bar: 20 µm. (**B**) Quantification of RBPMS-positive RGCs expressed as count of RGCs per mm^2^. (n = 4 mice per group). (**C**) β-III-tubulin (TUJ1) staining of RGC axon bundles and cell bodies in the retina. Scale bar: 20 µm (n = 4 mice per group). (**D**) Quantification of count of TUJ1-positive RGCs per mm^2^. (n = 4 mice per group). (**E**) Representative retinal whole-mounts from glaucomatous mice stained with NFH, showing the entire retinal surface reconstructed from Z-stacks spanning the full whole-mount thickness. Scale bar: 20 µm. (**F**) Quantification of NFH-positive RGC axon area. (n = 4 mice per group). (n = 4 mice per group). (**G**) Representative images showing that Oligo-2 immunoreactivity (red) in ONH. Scale bars: 50 µm. Nuclei are counterstained with Hoechst (blue). Scale bar: 50 µm. (**H**) Quantification of Oligo-2–positive cells per ONH area (mm^2^) (n = 4 mice per group). Graph bars correspond to DEX-vehicle (red), DEX-sitagliptin (blue), and untreated controls (white). Data are presented as mean ± SEM. * *p* < 0.05; ** *p* < 0.01. Abbreviations: GCL, ganglion cell layer; INL, inner nuclear layer; IPL, inner plexiform layer; ONL, outer nuclear layer; OPL, outer plexiform layer.

We observed a significant loss of both RNA-binding protein with multiple splicing (RBPMS) and ß-III-tubulin (TUJ1) in the DEX-vehicle compared to untreated controls (*p* < 0.05), thus indicating the decrease in cell bodies and axon bundles of RGCs. This effect was prevented by topical administration of sitagliptin (Figure 1A–D). In addition, a significant decrease in RGC axons marked by neurofilament heavy subunit (NFH) was found in the DEX-vehicle group when compared with the untreated control group (*p* < 0.001), thus indicating reduced axonal integrity, but the treatment with sitagliptin eye drops prevent this deleterious effect (Figure 1E,F). To further support these results, we performed a counting of RGCs using hematoxylin-eosin stained sections of the central retina in which the preventing effect on RGCs loss was clearly demonstrated (Supplementary Figure S2).

### 2.4. Sitagliptin Prevented Oligodendrocytes Loss in the ONH

The main function of mature oligodendrocytes is to generate myelin sheaths, which accelerate the conduction of nerve impulses and provide metabolic support for neuronal axons. We observed a significant loss of positive Oligo-2 cells in the DEX-vehicle compared to untreated controls (*p* < 0.05), and this effect was prevented by topical administration of sitagliptin (Figure 1G,H).

### 2.5. Sitagliptin Reduced the Astroglial Activation in the ONH and the Retina

**Figure 2:**
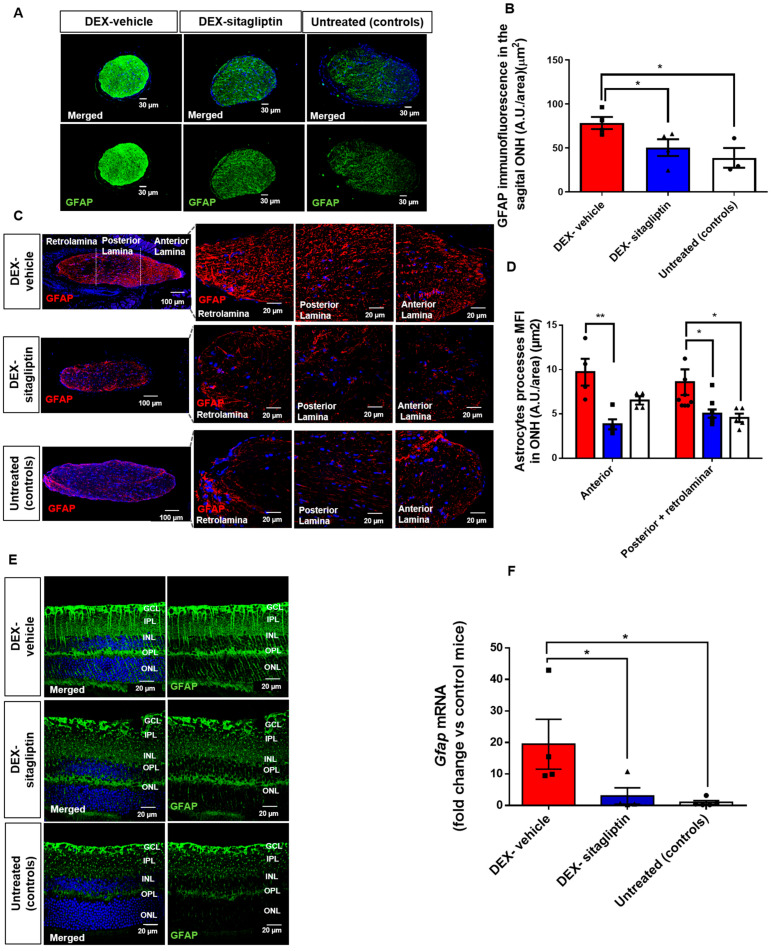
Sitagliptin reduced astroglial activation in the ONH and the retina. (**A**) Representative immunofluorescence images of the optic nerve head (ONH) stained with GFAP. Scale bar: 20 µm. (**B**) Quantification of GFAP relative expression in sagittal ONH sections (arbitrary units, A.U./area in µm^2^) (n = 4 mice per group). (**C**) GFAP immunofluorescence distribution in longitudinal ONH sections. Scale bar: 20 µm. (**D**) Quantitative comparison of GFAP intensity across ONH sectors (anterior, posterior, and retrolaminar). (n = 4 mice per group). (**E**) Representative images of retinal macroglial activation (GFAP). Scale bar: 20 µm. (**F**) mRNA levels of *Gfap* measured by qRT-PCR (n = 6 mice per group), represented as fold change relative to untreated controls. All data are presented as mean ± SEM. * *p* < 0.05; ** *p* < 0.01. Nuclei were counterstained with Hoechst (blue). Bars in panels (**B**,**D**,**F**) represent DEX-vehicle (red), DEX-sitagliptin (blue), and untreated controls (white). Abbreviations: GCL, ganglion cell layer; INL, inner nuclear layer; IPL, inner plexiform layer; ONL, outer nuclear layer; OPL, outer plexiform layer.

We observed an increase in the expression of GFAP in the ONH region in the DEX-vehicle group, in comparison with the control group, suggesting glial activation (Figure 2A,B). All regions of the ONH in the DEX-vehicle group exhibited immunoreactivity to GFAP, including both the anterior (AR) and posterior (PR) regions, as well as the retrolaminar region (RL) (Figure 2C,D). Topical administration of sitagliptin prevented glial activation in the AR, PR, and RL regions of the ONH, showing a significant reduction in the GFAP fluorescence intensity of astrocyte processes across all regions (*p* < 0.05) (Figure 2A–D).

In a glaucomatous retina, three types of glial cells, astrocytes, microglia, and Müller cells, can become activated. Immunofluorescence for GFAP revealed that in the retinas of untreated controls, positive staining was mainly localized in astrocytic cells located in the GCL layer. In mice with DEX-induced glaucoma, an up-regulation of retinal GFAP was observed to extend through all retinal layers, including the inner plexiform layer (IPL), inner nuclear layer (INL), outer plexiform layer (OPL), and some processes even extended up to the photoreceptor layer (ONL), thus indicating Müller cells activation (Figure 2E). We found an upregulation of mRNA levels of *Gfap* in DEX-induced glaucoma when compared with untreated controls. Treatment with eye drops of sitagliptin significantly inhibited retinal glial activation (Figure 2F).

### 2.6. Sitagliptin Reduced the Microglial Activation in the ONH

**Figure 3:**
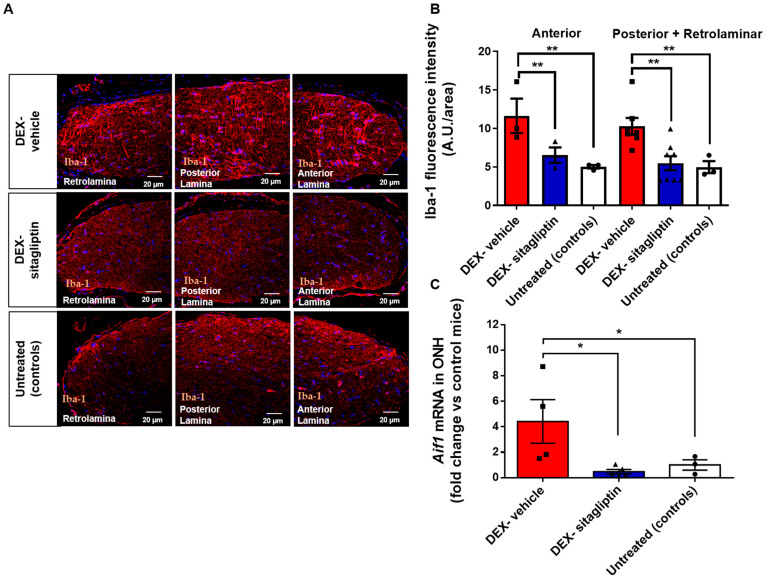
Sitagliptin reduces microglial activation in the ONH. (**A**) Representative immunofluorescence images of microglial distribution in adjacent transverse sections of the optic nerve head (ONH), including retrolaminar, anterior, and posterior regions, stained with Iba-1. Nuclei were counterstained with Hoechst (blue). Scale bar: 20 µm. (**B**) Quantification of Iba-1–positive microglial cells across ONH regions. (n = 4 mice per group). Bars represent DEX-vehicle (red), DEX-sitagliptin (blue), and untreated controls (white). (**C**) mRNA expression levels of Iba-1 (*Aif1* gene) measured by qRT-PCR (n = 6 mice per group), presented as fold change relative to untreated controls. Bars represent DEX-vehicle (red), DEX-sitagliptin (blue), and untreated controls (white). All results are shown as mean ± SEM. * *p* < 0.05; ** *p* < 0.01.

In the central nervous system, ionized calcium binding adaptor molecule (Iba-1) expression is confined to microglia and macrophages. The reduction in RGCs in experimental glaucoma models is linked to astrogliosis and axonopathy within the ONH, accompanied by upregulation of microglia. In DEX-induced glaucoma, we observed an upregulation of Iba-1 at the protein level in the ONH, suggesting microglial activation (Figure 3A,B). This upregulation was evident in both the central and peripheral regions of the ONH in the AR, PR, and RL areas in glaucoma compared to untreated controls. We also found that elevated expression of Iba1 protein coincided with the upregulation of *Aif1* mRNA expression (Figure 3C). Both *Aif1* mRNA expression and protein levels of Iba-1 were reduced in the animals treated with sitagliptin eye drops in all regions of the ONH (Figure 3A–C).

### 2.7. Sitagliptin Inhibits the Overexpression of Galectin-3 (MAC-2) and Gamma-Synuclein (γ-Synuclein) in the ONH

**Figure 4:**
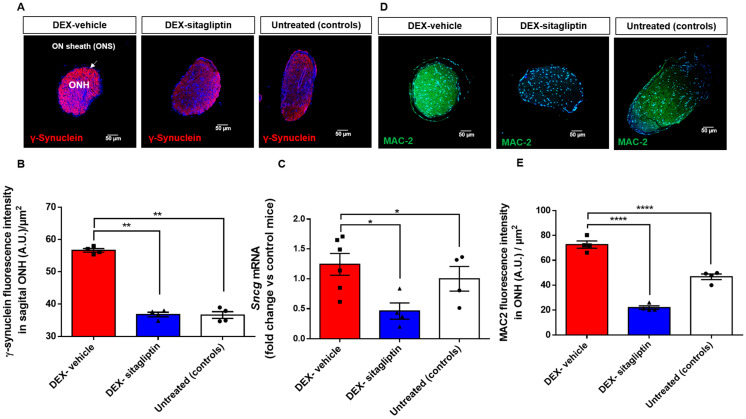
Sitagliptin inhibits the overexpression of galectin-3 and gamma-synuclein in the ONH. (**A**) γ-Synuclein immunofluorescence assay in sagittal ONH. Scale bars: 50 μm. (**B**) Quantification of γ-synuclein fluorescence intensity in sagittal ONH (A.U.)/µm^2^. (n = 4 mice per group). (**C**) mRNA levels of the *Sncg* gene by qRT-PCR. (n = 6 mice per group). (**D**) Galectin-3 (Mac-2) immunofluorescence in sagittal sections of ONH. Scale bars: 50 μm. (**E**) Quantification of extracellular Galectin-3 (MAC2) fluorescence intensity in sagittal ONH (A.U.)/µm^2^. (n = 4 mice per group). Nuclei were labeled with Hoechst (blue). Results are shown as mean ± SEM. * *p* < 0.05. ** *p* < 0.01. **** *p* < 0.001. Results are presented as fold change vs. control mice.

The accumulation of γ-synuclein, exhibiting a synucleinopathy phenotype, may contribute to neuronal death [19]. We found a significant mRNA overexpression of *Sncg*, as well as γ-synuclein fluorescence intensity in the ONH of DEX-induced glaucoma mice in comparison with the control group. Treatment with sitagliptin eyedrops was able to fully prevent this deleterious effect (Figure 4A–C).

Galectin-3 (Gal-3), also named MAC2, is a member of a carbohydrate-binding protein family involved in cell activation and inflammation [20,21]. Particularly Gal-3 plays a crucial role in microglial activation as an immunomodulatory mediator in response to inflammation and apoptosis. Gal-3 is expressed in various types of glial cells, including microglia, oligodendrocytes, and astrocytes [21,22,23,24]. We observed that Gal-3 was increased in the extracellular matrix of glial cells in the ONH in mice with DEX-induced glaucoma compared to untreated controls. Topical treatment with sitagliptin significantly reduced the expression of Gal-3 (Figure 4D,E).

### 2.8. Sitagliptin Decreased the Overexpression of Pro-Inflammatory Cytokines in the ONH

**Figure 5:**
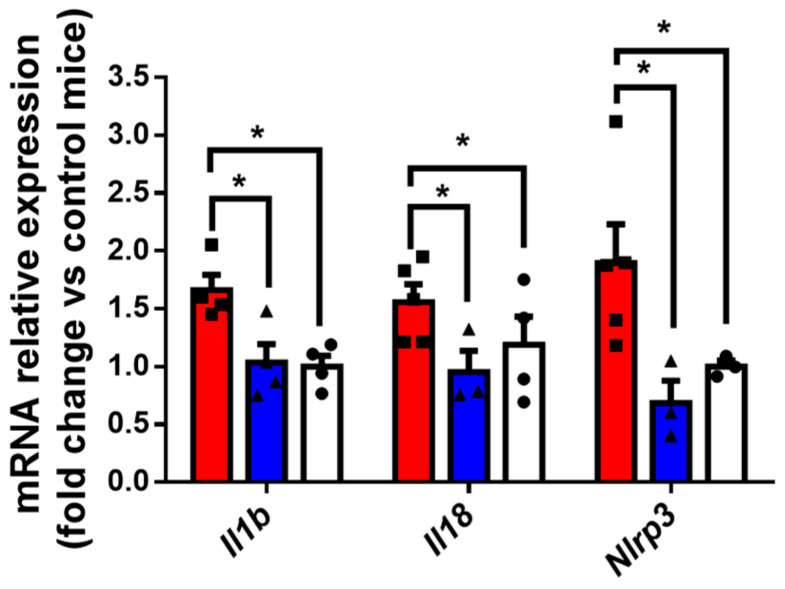
Sitagliptin decreased the overexpression of pro-inflammatory cytokines in the ONH. qRT-PCR expression analysis of pro-inflammatory cytokine *Il1-b*, *Il-18* and *Nlrp3* in ONH. (n = 6 mice per group). DEX-vehicle (red bars), DEX-sitagliptin (blue bars) and untreated controls (white bars). Results are presented as fold change vs. untreated control mice. Results are shown as mean ± SEM. * *p* < 0.05.

Inflammation is a well-recognized contributor to the development and progression of glaucoma [24]. We found that ONH from mice with DEX-induced glaucoma presented a significant increase in mRNA levels of the NOD-like receptor family, pyrin domain-containing 3 (*Nlrp 3*), which is responsible for the multiprotein complex triggering inflammatory responses, as well as *Il 1b* and *Il 18* in comparison with controls. Again, sitagliptin was able to abrogate this overexpression (Figure 5).

## 3. Discussion

A significant proportion of patients with glaucoma have normal IOP [2]. Moreover, the achievement of low IOP is often not enough to prevent glaucoma progression and the disease may progress despite normalization of the IOP [25,26]. For all these reasons, glaucoma can be considered a primary optic neuropathy that makes RGCs more vulnerable not only to elevated IOP but also to other risk factors that still remain to be elucidated [27,28]. These fundamentals support the current concept that neuroprotection plays a key role in treating glaucoma [26,29]. In the present study, we have found that topical administration (eye drops) of sitagliptin exerts a potent neuroprotective effect on RGCs and ONH in a mouse model of glaucoma induced by periocular administration of glucocorticoids. The effectiveness in neuroprotection of sitagliptin eye drops was based on the significant prevention of RGCs and neurofilament loss, as well as by the dramatic downregulation of glial activation in both the neuroretina and ONH. Additionally, sitagliptin inhibited the overexpression of galectin-3 and gamma-synuclein in ONH, two significant mediators of the neurodegenerative process in glaucoma. These findings suggest that eyedrops of sitagliptin alone or combined with IOP lowering agents could be a new neuroprotective approach for treating glaucoma.

RGCs apoptosis and axonal loss within the inner retina are the earliest manifestation of glaucoma and exhibit a direct correlation with the clinical severity of the disease [27,28]. The pathophysiology of RGC apoptosis is complex, involving both mechanical and vascular components [28]. In the present study we have found that ocular topical administration of sitagliptin prevented the loss of RGCs measured by both antibodies against RNA-binding protein with multiple splicing (RBPMS) and anti β-III-Tubulin, two robust methods for identifying RGCs [30,31].

The underlying molecular mechanisms involved in RGC apoptosis in glaucoma remain to be fully elucidated, but inflammation plays an important role [24,28]. Microglia and macroglia are the cell types involved in inflammatory responses within the retina. Under pathological conditions, these glial cells become reactive, lose their homeostatic functions of trophic and metabolic support, and gain neurotoxic properties that trigger inflammatory-mediated neurodegeneration [24,32]. We have found that topical administration of sitagliptin inhibited the overexpression of both macro and microglial activation that exists in the neuroretina and in the ONH, which was associated with a downregulation of NLRP3 and proinflammatory cytokines. In addition, sitagliptin abrogated the overexpression of galectin-3 (Gal-3) that exists in the ONH in the experimental model of steroid-induced glaucoma. This is an interesting observation because Gal-3 is currently considered a rising star in modulating microglia activation under conditions of neurodegeneration [33,34]. In fact, Gal-3 could potentially stimulate NLRP3 inflammasome and NF-kB pathways, resulting in the release of pro-inflammatory cytokines [21]. Moreover, Gal-3 deletion has been shown to be neuroprotective in different models of neurodegeneration [21,33,34]. For all these reasons, the downregulation of galectin-3 can be considered an essential mechanistic finding to understand the neuroprotective effects of sitagliptin.

Glaucoma is distinguished from other optic neuropathies by its selective loss of RGC axons. There is cumulating evidence showing that axon dysfunction and degeneration are the key insult that drives glaucomatous neurodegeneration [23,35,36]. The capacity of the neurofilament cytoskeleton to dismantle and adjust in reaction to local alterations in the neuronal environment is crucial for meeting axonal energy requirements. In pathological conditions such as glaucoma, the loss and dephosphorylation of neurofilament will deprive metabolic routes of essential substrate for axonal energetics, thereby increasing the susceptibility of axons to injury. Neurofilament heavy subunit (NFH) is one of these cytoskeleton proteins [36,37]. In the present study, we have observed a significant loss of NFH, which was prevented by topical administration of sitagliptin. Previously, neurofilament loss and dephosphorylation have been shown in animal models of optic nerve injury, including a glaucoma model [37,38,39], but to the best of our knowledge, this has not previously reported in the glaucoma model induced by dexamethasone.

Oligodendrocytes together astrocytes are the most abundant cells types of neuroglia and by generating myelin among other actions play a critical role in axonal structure and function [40]. The loss of myelin and the impairment of oligodendrocytes fundamentally alters the neuron, rendering demyelinated axons susceptible to energetic failure and the accumulation of intracellular calcium, thereby driving subsequent degeneration [41,42,43]. In the setting of glaucoma, the death of oligodendrocytes in the optic nerve has been reported in several experimental models [44] but, to the best of our knowledge, this is the first report showing a significant loss of oligodendrocytes in glaucoma induced by dexamethasone. More importantly, topical administration of sitagliptin prevents the loss of oligodendrocytes.

Gamma-synuclein can be considered a member of the Bcl-2 apoptosis family, and its overexpression participates in the pathogenesis of glaucoma by facilitating the disintegration of neurofilament networks, by activating astrocyte phagocytosis in the ONH and inhibiting the optic nerve regeneration [45,46]. Our findings support that these pathogenic events are also present in the DEX-induced glaucoma model and that they are abrogated by the topical administration of sitagliptin. Therefore, the downregulation of gamma-synuclein can be added to the underlying mechanisms of action by which sitagliptin exerts its beneficial action in glaucoma.

The mechanistic effects of eyedrops of sitagliptin above summarized can be produced by both GLP-1R activation due to the enhancement of retinal amount of GLP-1 as a result of inhibiting its degradation or through direct mechanisms unrelated to GLP-1. These latter pathways have been suggested by we [13] and others [10,11,12]. In addition, we have recently reported that sitagliptin prevents the hyperpermeability induced by diabetic milieu in both RPEs and HRECs cultures independently of GLP-1 [9]. The experimental mouse model used in the present study is based on steroid-induced ocular hypertension by periocular injections of dexamethasone. Although the mechanisms by which glucocorticoids lead to glaucoma are not well understood, it has been reported that trabecular meshwork express glucocorticoid receptors and their activation by exogenous steroids, such as dexamethasone, decrease the phagocytic ability of trabecular meshwork cells and downregulate metalloproteinases (i.e., MMP1), thus leading to extracellular matrix deposition, particularly in the juxtacanalicular tissue and along the inner wall endothelium of Schlemm’s canal. The result is increased resistance to aqueous flow and a consequent rise in IOP [18,47]. Steroid-induced glaucoma is a major clinical problem, especially with the use of intravitreal glucocorticoid therapies to treat diabetic macular edema. This adverse effect is currently treated with IOP-lowering pharmaceutical therapies and patients are switched to intravitreal anti-VEGF agents if they have not already received this type of treatment. However, intravitreal administration of glucocorticoids could be the last chance for a significant proportion of patients. Our results open up a potential new strategy using sitagliptin in combination with IOP-lowering drugs as an adjuvant treatment to prevent RGCs and ONH neurodegeneration, thus allowing a more extended and prolonged treatment. In addition, it should be noted that one of the deleterious effects of repeated anti-VEGF injection is also neurodegeneration of the healthy retina [48]. In this regard, topical administration of sitagliptin combined with anti-VEGF agents could also be considered in order to prevent this potential adverse effect.

Potential study limitations of the present study may include the reduced sample size of mice used, the histological analysis focused on central retina, the lack of functional examinations, and the contribution of GLP-1R activation to the observed beneficial results. The use of only female mice might also be considered a limiting factor. However, although a growing body of evidence demonstrates a protective role of estrogen in glaucoma, standardized studies are needed to further elucidate the roles of estrogen and testosterone in glaucoma risk and progression [49]. Moreover, the model used in the present study (dexamethasone-induced glaucoma) seems not be influenced by the sex [50,51].

In summary, current therapeutic strategies aimed at lowering IOP are not sufficient to prevent glaucoma-related blindness, and therefore, new effective approaches focusing on neuroprotection are needed. We provide evidence that topical administration of sitagliptin exerts a potent neuroprotective action in experimental dexamethasone-induced glaucoma. Our results open a new avenue to address neurodegeneration in glaucoma, particularly in cases secondary to intravitreal steroid administration.

## 4. Materials and Methods

### 4.1. Animals

Thirty female C57BL/6J mice (39 weeks old) from Charles River Laboratories Inc. (Calco, Italy) were randomly housed in pairs in Tecniplast GM-500 cages (Buguggiate, Italy) under controlled environmental conditions with specific humidity (50–60%), temperature (22 ± 2 °C), and light/dark cycles. They had access to filtered water and food ad libitum. Body weight was monitored weekly throughout the duration of the experiment. All accomplished experiments with animals were adjusted in compliance with the European Community (86/609/CEE) and adhered to the guidelines set by the ARVO Statement for the Use of Animals in Ophthalmic and Vision Research and were approved by the Animal Care and Use Committee of Vall d’Hebron Research Institute (CEEA 54/21).

### 4.2. Dexamethasone Periocular Administration, Multimodal Imaging, and Topical Treatment with Sitagliptin

The model of glaucoma induced by periocular administration of dexamethasone (DEX; Laboratorios ERN, Barcelona, Spain) was used as previously reported [52,53]. For this experiment, 3 groups of mice (41 weeks old) received every week for 5 weeks a bilateral injection in the conjunctival fornix of (1) 20 µL DEX 8 mg/mL (n = 10); (2) 20 µL of vehicle (phosphate-buffered saline (PBS) pH 7.4; n = 10); and (3) or untreated as controls (n = 10). At week 43, DEX-treated mice received topical ocular administration of 5 µL sitagliptin monohydrate phosphate (10 mg/mL; Y0001812, Merck KGaA, Darmstadt, Germany) (n = 10) or vehicle (PBS) eye drops (n = 10) twice daily for 3 weeks (Supplementary Figure S3). On the final day, a drop of sitagliptin or vehicle was administered to the eyes 1 h prior to euthanasia. Age-matched mice (n = 10) were used as a control group. In vivo multimodal imaging was performed as previously described [54] (Supplementary Information).

Sitagliptin was selected because in our hands it has a slightly but consistently more powerful action in protecting the neurovascular unit as a whole (neuroprotection and vascular leakage inhibition) than saxagliptin.

### 4.3. Intraocular Pressure (IOP) Measurement

Non-invasive measurement of intraocular pressure (IOP) was conducted using the TonoLab rodent rebound tonometer (TonoLab; Icare, Helsinki, Finland), following the manufacturer’s guidelines. Mice were anesthetized with 1% isoflurane and 1.0–1.5 L/min oxygen for 2 min in an anesthesia chamber, according to published protocols [55,56]. Isoflurane anesthesia may lower absolute IOP values; however, measuring within the first 3 min after induction is considered valid [56,57], as was done in our study, and is essential for obtaining reliable and reproducible data. Experimental validity is preserved because all groups were subjected to the same anesthetic protocol, ensuring that any anesthetic-related reduction in IOP affects all animals equally. This approach allows true biological differences between groups to be detected more clearly due to reduced noise and a lower standard deviation. They were gently restrained on a height-adjustable platform while maintaining anesthesia through a facemask. The IOP measurements were performed weekly between 9 and 10 am, ensuring the probe was aligned perpendicular to the central cornea. The tonometer automatically took six measurements, excluding the highest and lowest readings, and calculated the mean of the four intermediate readings from three consecutive measurements.

### 4.4. Immunofluorescence of the Neuroretina, ONH Sections and Retinal Wholemounts

Immunofluorescence analyses were performed on paraffin sections as described in Supplementary Material. Antibodies are listed in Supplementary Information (Tables S1 and S2). Images were captured using a confocal laser scanning microscope (FV1000; Olympus Laser Scanning Confocal Microscope, Olympus Corporation, Tokio, Japan). Five fields were selected for analysis comprising from the lateral margins of the optic nerve head to a distance of up to 300 μm within the central retina. This distance served as an objective anatomical reference to ensure the region selection consistency across all samples. Each field was analyzed at a resolution of 1024 × 1024 pixels. Immunofluorescence intensities were quantified using FIJI ImageJ software (version 1.8, U. S. National Institutes of Health, Bethesda, MD, USA). Background fluorescence was measured from unstained regions within each image and subtracted from the raw fluorescence values. This approach ensured that the quantification reflected only the specific signal while minimizing the influence of nonspecific background. Data were analyzed by investigators who were masked to the group assignments.

### 4.5. Quantification of RGCs in Retinal Tissues

To assess the expression of the RNA-binding protein with multiple splicing (RBPMS) and ß-III-tubulin (TUJ1) in RGCs, immunofluorescence was performed on paraffin-embedded samples, as previously reported [58,59]. RBPMS-positive and TUJ1-positive RGCs were quantified and expressed as the number of RGCs per mm^2^. In addition, cells of RGC layer were also quantified on Hematoxilin & Eosin-stained sections (Supplementary Material). To minimize bias, all procedures and analyses were conducted under blinded conditions.

### 4.6. Quantification of Fluorescent Immunostainings in Optic Nerve Head (ONH)

Measurements were performed on sagittal sections of the ONH or in regions of interest (ROIs) within transverse sections of the ONH, categorized as anterior, posterior, and retrolaminar. Images were acquired using the laser scanning confocal microscope (FV1000 Laser Scanning Confocal Microscope Olympus, Hamburg, Germany) and fluorescence intensity was measured using ImageJ (version 1.8, U. S. National Institutes of Health, Bethesda, MD, USA).

### 4.7. Macroglial Activation

Astrocyte processes were detected using the glial fibrillary acidic protein (GFAP) antibody in retinal sections, ONH cross sections, and whole mounts. For quantification of GFAP immunofluorescence in ONH sections, three sections per mouse were analyzed, with background fluorescence subtracted. Micrographs were captured from the anterior, posterior, and retrolaminar regions of each ONH section from every mouse in each experimental group, providing a comprehensive quantification of the entire optic nerve, using a laser scanning confocal microscope (FV1000).

### 4.8. Microglial Molecular Signature

Microglia were analyzed using Iba1 antibody staining to quantitatively assess microglial activation and distribution in different regions of the ONH (n = 4). The mean intensity was determined by outlining the region of interest (ROI) using a predefined mask, applying the same threshold value across all images. The mean intensity within each ROI was then automatically measured. All analyses were performed by researchers blinded to the experimental groups (Supplementary Information).

### 4.9. Galectin 3 (MAC-2) and Gamma-Synuclein (γ-Synuclein)

Gal-3 (MAC-2) and γ-Synuclein intensity levels were quantified in sagittal ONH sections (n = 4) and the results were expressed in fluorescence intensity per square millimeter. A minimum of two ONH sections were analyzed from each mouse. To minimize bias, all procedures and analyses were conducted under blinded conditions.

### 4.10. Oligodendrocyte Transcription Factor 2 (Oligo-2)

We designed a macro that allows automated analysis of multichannel composite images. For each image (n = 4), the macro selected the channel dedicated to Oligo-2 staining, set a user-defined fluorescence threshold, created a mask, and counted Oligo-2 positive cells with the “Analyze Particles” function. A similar process was applied separately to determine the ONH areas. Finally, the macro calculated the Oligo-2-positive cell count per ONH area in mm^2^.

### 4.11. RNA Isolation and qRT-PCR

Total retinal RNA and ONH RNA from mouse retinas were extracted and transcribed as previously described (12). Quantitative RT-PCR was conducted in 384-well plates (ThermoFisher Scientific, Waltham, MA, USA) with SYBR Green PCR Master Mix (4309155, Applied Biosystems, Warrington, UK) following standard conditions. Each sample was analyzed in triplicate, and the relative fold change in gene expression levels was calculated using the formula 2-ddCt, with β-actin (ActB) and β2-microglobulin (B2m) serving as the internal controls. The primer pairs used for qRT-PCR are listed in Supplementary Table S3.

### 4.12. Statistical Analysis

Statistical analysis was performed using GraphPad Prism 10 (GraphPad Software, Boston, MA, USA). The data were expressed as mean ± standard error of the mean (SEM). Differences among groups were analyzed by one-way ANOVA, followed by the Bonferroni’s multiple comparison post hoc test. A *p*-value < 0.05 was considered statistically significant.

## 5. Conclusions

The current treatment strategies focused on lowering intraocular pressure remain insufficient to halt the progression of glaucoma-related neurodegeneration. Our study provides compelling evidence that topical sitagliptin confers significant neuroprotection in a dexamethasone-induced glaucoma model. These findings highlight sitagliptin as a promising therapeutic candidate for preventing steroid-induced glaucomatous neurodegeneration.

## Data Availability

The data sets used and/or analyzed during the current study are available from the corresponding author on reasonable request.

## Supplementary Materials

The following supporting information can be downloaded at: https://www.mdpi.com/article/10.3390/ijms27010048/s1.

## Abbreviations

ActB	β-actin
AR	anterior region of ONH
ARVO	Association for Research in Vision and Ophthalmology
B2m	β2-microglobulin
DEX	dexamethasone
DPP-IV	dipeptidyl peptidase IV
DR	diabetic retinopathy
GFAP	glial fibrillary acidic protein
GLP-1	Glucagon-Like Peptide
INL	inner nuclear layer
IPL	inner plexiform layer
IOP	intraocular pressure
MAC-2	Galectin 3
NFH	neurofilament heavy subunit
NLRP3	NOD-like receptor pyrin domain-containing protein 3
ONH	optic nerve head
ONL	photoreceptor layer
Oligo-2	Oligodendrocyte transcription factor 2
OPL	outer plexiform layer
POAG	primary open-angle glaucoma
PR	posterior region of ONH
RBPMS	RNA-binding protein with multiple splicing
RL	retrolaminar region of ONH
ROIs	regions of interest
RGCs	retinal ganglion cells
TUJ1	β-III-tubulin
γ-Synuclein	gamma-synuclein
IL-1β	Interleukin-1β
IL-18	Interleukin-18
