# Supplementary material for: Topical Administration of Sitagliptin Prevents Retinal Neurodegeneration in a Model of Glaucoma Induced by Dexamethasone
Source: Int J Mol Sci. 2025 Dec 20;27(1):48. doi: 10.3390/ijms27010048 (PMC12785737; doi:10.3390/ijms27010048)
Supplement: Supplementary file 1 [file ijms-27-00048-s001.zip › ijms-3976550-supplementary.pdf]

Supplementary Table S1. Primary antibodies used for immunofluorescence assay

Target Molecule	Clone	Blocking Conditions	Dilution	Manufacturer
Galectin 3 (Mac2)	Mouse monoclonal	Protein Block Serum-Free X0909, Dako	1/100	Abcam [A3A12] (ab2785)
GFAP	Rabbit polyclonal	10% NGS, 1% BSA in PBS pH 7.4	1/200	Abcam (ab7260)
Iba-1	Rabbit polyclonal	5% NGS, 0.1% Triton X-100 in PBS pH 7.4	1/100	Wako (019-19741)
NFH	Chicken polyclonal	Protein Block Serum-Free X0909, Dako	1/150	Abcam (ab4680)
Gamma-Synuclein (γ-Synuclein)	Rabbit polyclonal	Protein Block Serum-Free X0909, Dako	1/100	Abcam (ab55424)
RBPMS	Rabbit polyclonal	Protein Block Serum-Free X0909, Dako	1/200	Thermofisher Scientific (PA5-31231)
ß-III-tubulin (Tuj1)	Mouse monoclonal	Protein Block Serum-Free X0909, Dako	1/200	Biolegend (801201)
Oligo-2	Rabbit monoclonal	Protein Block Serum-Free X0909, Dako	1/100	Abcam (ab109186)

NGS: Normal goat serum; BSA: Bovine serum albumin; PBS: Phosphate-buffered saline

Supplementary table S2. Secondary antibodies used for immunofluorescence assay

Secondary antibodies	Clone	Dilution	Manufacturer
Alexa Fluor® 594	Goat anti-rabbit	1/600	ThermoFisher Scientific (A-11012)
Alexa Fluor® 488	Goat anti-rabbit	1/600	Abcam (ab150081)
Alexa Fluor®405	Goat anti-chicken	1/600	ThermoFisher Scientific (A48260)
Alexa Fluor® 594	Goat anti-chicken	1/600	Abcam (ab150176)
Alexa Fluor® 488	Goat anti-mouse	1/600	Abcam (ab150113)
Alexa Fluor® 594	Goat anti-mouse	1/600	ThermoFisher Scientific (A-11032)

Supplementary table S3. Primers used for qRT-PCR

Primers	Gene ID	Nucleotide sequence	
		**Forward (5′-3′)**	**Reverse (5′-3′)**
B2m	12010	GTATGCTATCCAGAAAACCC	CTGAAGGACATATCTGACATC
Actb	11461	CTAAGGCCAACCGTGAAAG	CAGTATGTTCGGCTTCCCATTC
Aif (Iba1)	11629	AGCCAGACGAACCCTCTGAT	CCACATCAGCTTTTGAAATCTCCTC
Gfap	14580	TCCTTGTCTCGAATGACTCC	CTGTGCAAAGTTGTCCCTCT
Sncg	6623	TGGGCACCAAAACCAAGGAGA	CTGTGTTGACGCTGCTGAC
Il1b	16176	GCAACTGTTCCTGAACTCAACT	ATCTTTTGGGGTCCGTCAACT
Il18	6173	AAATGGAGACCTGGAATCAG	CCTCTTACTTCACTGTCTTTG
Nlrp3	216799	ATCAACAGGCGAGACCTCTG	GTCCTCCTGGCATACCATAGA

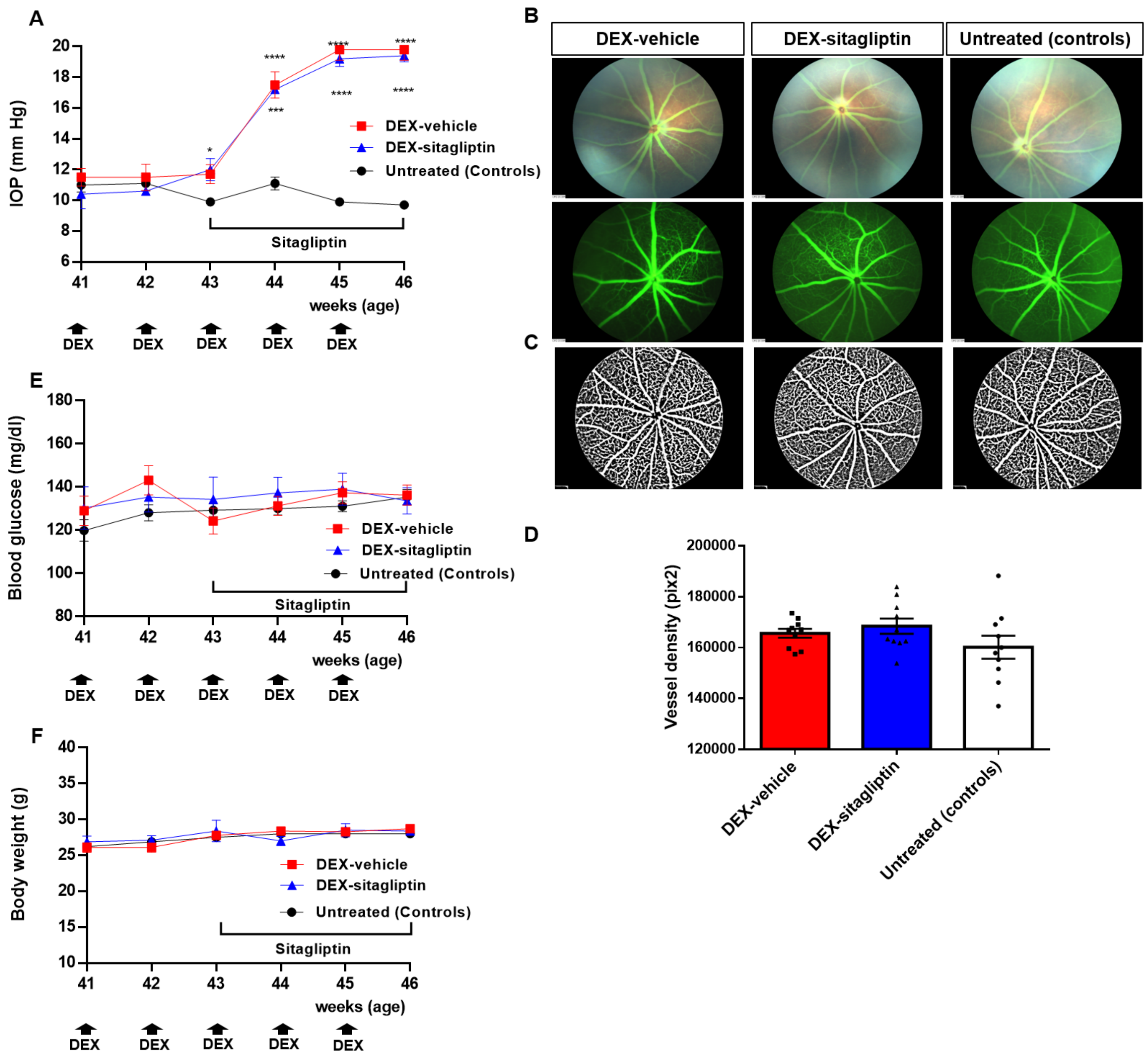

Supplementary Figure S1 (A) Non-invasive intraocular pressure (IOP) measurements in DEX-induced glaucoma mice treated with DEX-vehicle (red squares, n=10) or DEX-sitagliptin 10 mg/ml (blue triangles, n=10), compared with untreated control mice (black circles, n=10), recorded at 41–46 weeks of age. Statistical differences were assessed between DEX-vehicle and untreated controls (*$p < 0.05$; ***$p < 0.01$; ****$p < 0.001$). **(B)** Representative fundoscopy images (upper panel) and fundus fluorescence angiography (middle panel) showing vascular arborisation in each experimental group (n=10 mice per group). **(C)** Corresponding binary masks generated from fluorescence angiography images using ImageJ (lower panel) **(D)** Quantification of vessel density (pixel^2) from fundus fluorescence angiography images (n=10 mice per group). **(E)** Blood glucose levels in DEX-induced glaucoma mice treated with DEX-vehicle (red squares, n=8) or DEX-sitagliptin 10 mg/ml (blue triangles,

n=10), compared with untreated controls (black circles, n=10). **(F)** Body weight of DEX-induced glaucoma mice across the same experimental groups: DEX-vehicle (red squares, n=10), DEX-sitagliptin 10 mg/ml (blue triangles, n=10), and untreated controls (black circles, n=10).

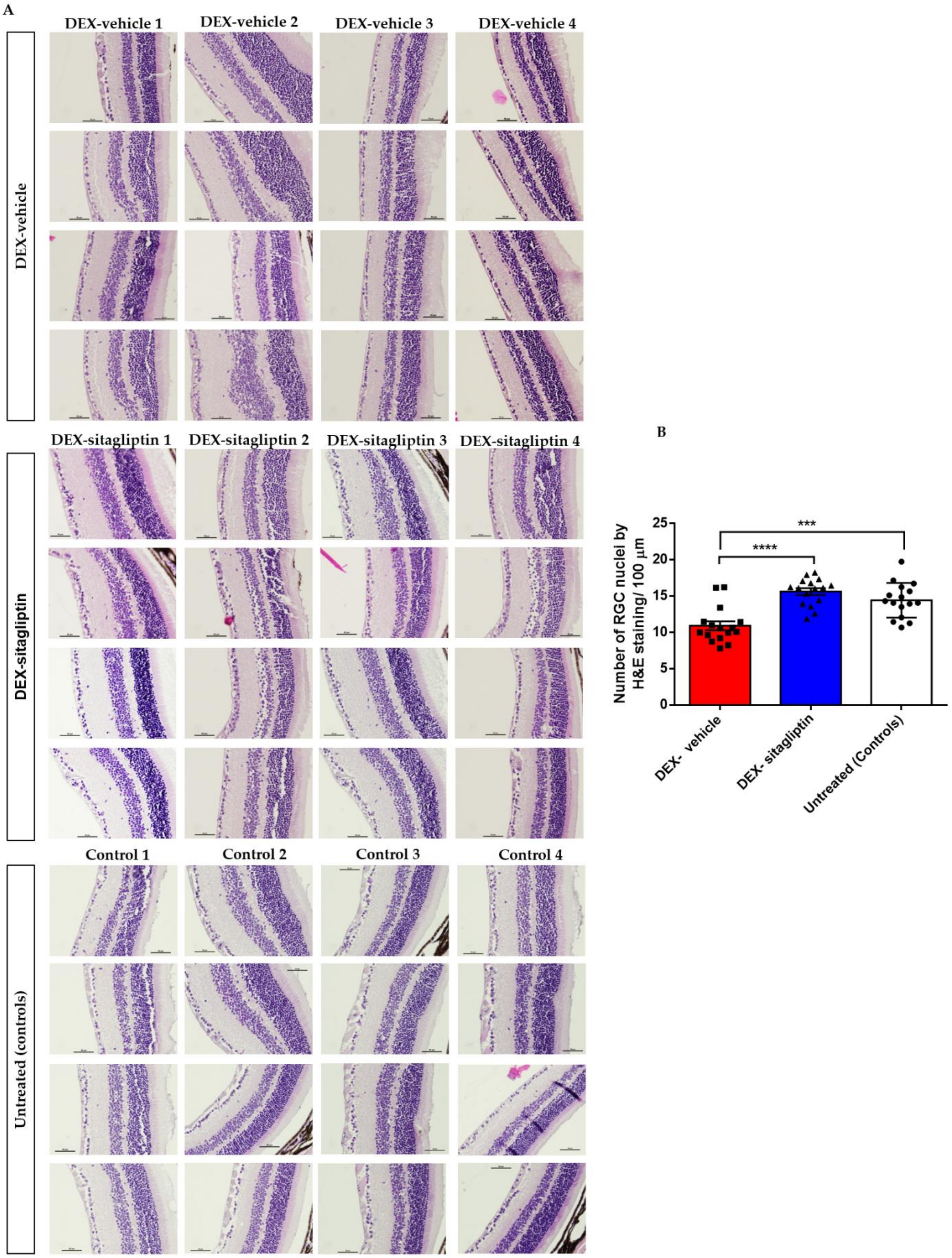

Supplementary Figure S2. Effects of sitagliptin on retinal ganglion cell (RGC) density in Dexa-treated mice.(A) H&E-stained retinal sections showing the RGC layer in untreated controls, Dexa-vehicle, and Dexa-sitagliptin groups. **(B)** Quantification of RGC nuclei per 100 μm in the central retina (n = 16 mice per group). Data are presented as mean ± SEM. RGC density is shown for untreated controls (white bars), Dexa-vehicle (red bars), and Dexa-sitagliptin (blue bars). Dexa-vehicle treatment caused a 24% reduction in RGC density compared with untreated controls (10.92 ± 0.62 vs. 14.45 ± 0.60 cells/100 μm), whereas Dexa-sitagliptin partially restored RGC density, showing an 8% increase relative to untreated controls (15.65 ± 0.47 cells/100 μm). ***p < 0.001 (Dexa-vehicle vs. untreated controls); ****p < 0.0001 (Dexa-vehicle vs. Dexa-sitagliptin).

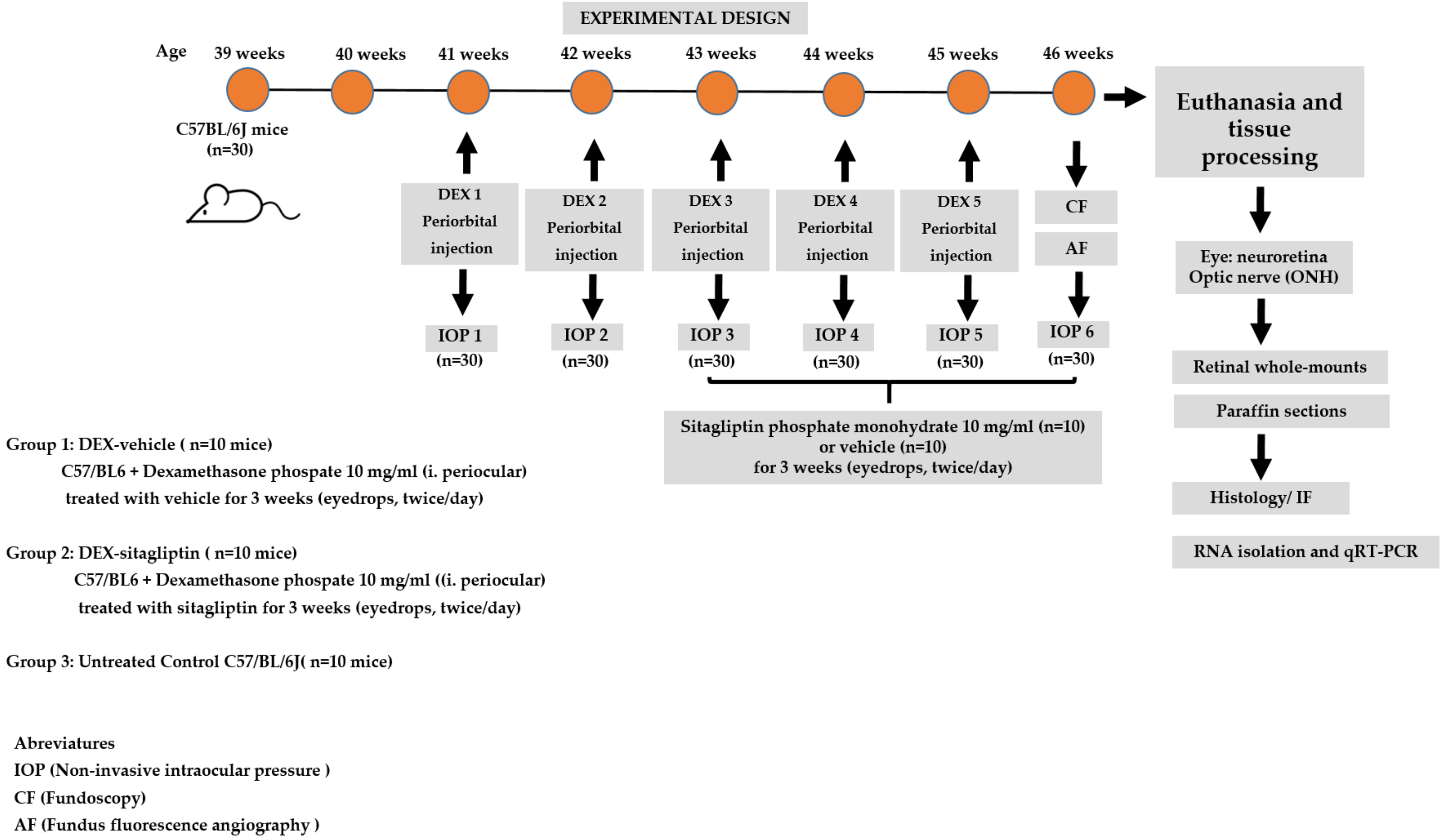

Supplementary Figure S3. Scheme of the experimental protocol

Supplementary Methodology

1. Supplementary Materials and Methods

1.1 Animals

All animal procedures complied with European Community Animal Care guidelines (86/609/CEE) and approved by the Use Committee of VHIR (Vall d'Hebron Research Institute, Barcelona, Spain) approval code 54/21; approval date of 2022. The study also adhered to the Association for Research in Vision and Ophthalmology (ARVO) recommendations for the use of laboratory animals.

1.2 Multimodal in vivo imaging

Tropicamide eyedrops (Colircusi Tropicamida® 10 mg/mL, Alcon Laboratories, El Masnou, Spain) were used to dilate the pupil, and 2% Methocel gel (OmniVision, Germany) was applied to the cornea to facilitate contact with the lens. At weeks 42 and 46, at the beginning and end of the treatment, animals underwent funduscopy and fundus fluorescence angiography (FA) using the Micron III platform (Phoenix Micron, USA). After anesthesia and pupil dilation, 10 μL/g sodium fluorescein (0.1% Fluorescein Oculos, Thea Laboratories, S.A. Barcelona, Spain) was administered intraperitoneally, and using a green filter, eye fundus images from the central retina were captured. All procedures were performed under general anesthesia with a mixture of 2% isoflurane (Arrane®, Baxter Laboratories, Victoria, Australia) / 1% O2. A heating pad was utilized to maintain a stable body temperature.

1.3 Tissue collection and processing

Mice were intraperitoneally injected as described (54) with 0.2 mL of anesthesia (a solution containing 1 mL of ketamine from GmbH, Hameln, Germany, and 0.3 mL of xylazine from Laboratorios Calier S.A., Barcelona, Spain) and then transcardially perfused with 4% paraformaldehyde (PFA; sc-281692, Santa Cruz Biotechnology, Dallas, TX, USA). The mice were euthanized, and their ocular globes were immediately removed. The retina from the right eye was snap-frozen in liquid nitrogen for mRNA extraction. The left eye and optic nerve heads (ONH) sections were fixed in 4% PFA for 6 hours and embedded in paraffin blocks for the retinal sections. Subsequently, 4 μm thick sections were cut with a microtome, with three sections placed on positively charged slides and stored at 4 °C. Six central retinal sections were used for immunofluorescence analysis. For retinal wholemounts, eyeballs were fixed in 4% PFA at 4 °C for 5 hours.

1.4. Immunostaining

For the immunofluorescence analysis, paraffin-embedded slides were heated at 65°C for 1 hour, deparaffinized in xylene, and rehydrated in a graded ethanol series. In both cases, sections and wholemounts were washed with PBS pH 7.4 (PBS; Biowest, Labclinics, Barcelona, Spain), fixed in ice-cold acid methanol for 1 minute at -20 °C, and washed with 0.01 M PBS at pH 7.4. Next, the sections were immersed in an antigen retrieval solution (sodium citrate 10 mM, pH 6.0) and heated in a pressure cooker at 150 °C for 4 minutes. Both sections and wholemounts were blocked with the blocking solution (see Table 1) for 1 hour at room temperature. All samples were then incubated overnight at 4 °C with the corresponding primary antibodies described in Supplementary Table S1. Some sections and wholemounts that underwent the same procedures except for the primary antibody incubation served as negative controls. The following day, the samples were washed twice in PBS and then incubated for 1 hour in darkness at room temperature along with the secondary antibody sections (Supplementary Table S2). After incubation, the sections were washed in PBS, counterstained with Hoechst 33342 (bisbenzimide) (14533, ThermoFisher Scientific, Waltham, MA, USA) at a dilution of 1:500, and coverslipped using a mounting

solution (Prolong Gold antifade reagent P36930 InvitrogenTM, Thermo Fisher Scientific, Eugene, OR, USA). Subsequently, images were captured using a confocal laser scanning microscope (FV1000; Olympus Laser Scanning Confocal Microscope, Olympus Corporation, Shinjuku, Japan). Five fields were selected for analysis in the region extending from the lateral margins of the optic nerve head to a distance of up to 300 μm within the central retina. This distance was used as an objective anatomical reference to ensure consistency in the location of the regions analyzed across all samples from each section were analyzed. The same locations and number of fields were measured in all neuroretinas at 60x and 40x magnification for the wholemounts, all at a resolution of 1024 x 1024 pixels. Immunofluorescence intensities were quantified using FIJI ImageJ software (version 1.8, U. S. National Institutes of Health, Bethesda, MD, USA).

The analysis involved determining the percentage of RBPMS-immunoreactive somas and the percentage of RGC axon bundles and cell bodies labeled with ß-III-tubulin (TUJ1) per area. The density of RBPMS-positive cells per unit area was quantified in terms of cells per square millimeter using FIJI-ImageJ software. For quantification, a minimum of two retinal sections were collected from each eye. Additionally, the cross-sectional area of RGC axons was quantified in retinal wholemounts using neurofilament heavy chain (NFH) immunostaining and with FIJI-ImageJ software, which allows measurement of individual cross-sectional areas of each identified RGC axon providing quantitative data on axon size and integrity. For each retina, multiple regions of interest (ROIs) were systematically selected to represent the entire retinal surface, using Z-stacks encompassing the full thickness of the wholemount. ROIs were chosen to avoid areas with tissue damage or artifacts. Within each ROI, NFL immunofluorescence intensity was measured after subtracting background fluorescence, providing a quantitative assessment of axonal density. All analyses were performed by researchers blinded to the experimental groups to prevent bias.

1.5. *H&E stained retinal preparations*

Eyeballs were harvested and fixed in 4% paraformaldehyde (PFA) at 20 °C for 5 h and subsequently processed for paraffin embedding. Serial sections (5 μm) were obtained, mounted on glass slides, and stained with hematoxylin and eosin (H&E) (Hematoxylin: Sigma-Aldrich H3136; Eosin: Sigma-Aldrich 318906). These sections were stained with H&E at room temperature for 10 minutes. After staining, sections were coverslipped and examined using a Nikon Eclipse Ts2R light-transmission microscope. Retinal ganglion cell (RGC) density was quantified by counting the number of nuclei within the RGC layer in the central retina over a standardized length of 300 μm (located 200–500 μm from the optic nerve head). Image analysis was performed using FIJI/ImageJ software (version 1.8, National Institutes of Health, Bethesda, MD, USA). Cells with clearly identifiable nuclei in the RGC layer were manually marked using the “Point Tool.” For each animal, four retinal images were analyzed, resulting in a total of 16 images per experimental group. Final RGC density values were expressed as the number of RGC nuclei per 100 μm.

1.6. *Microglial molecular assay*

To quantitatively assess microglial activation and distribution in different regions of the ONH, microglia were analyzed using Iba1 antibody staining. ROIs were divided into anterior, posterior, and retrolaminar areas. The mean intensity was determined by outlining the area with a mask using the same threshold value in each image, and then the mean intensity within these areas was automatically measured using Fiji ImageJ software.

In the whole-mount samples, the total numbers of microglial cells were quantified by calculating the Iba1-positive cells per area in each image using Fiji ImageJ software.

1.7. RNA isolation and qRT-PCR

Total retinal RNA and ONH RNA from mouse retinas were diluted in 140 μL and 80 μL of TRIzol reagent (15596018, InvitrogenTM, Carlsbad, CA, USA) for RNA extraction, respectively. To eliminate genomic contamination, neuroretinas were treated with DNase (18068015, ThermoFisher Scientific, Waltham, MA, USA) and then purified using an RNeasy MinElute column (74106, Qiagen, Hilden, Germany). The RNA concentration and integrity were assessed using a NanoDrop spectrophotometer (ThermoFisher Scientific). A minimum of 400 ng of RNA was reverse-transcribed into complementary DNA (cDNA) in a T100 Thermal Cycler (Bio-Rad, Hercules, CA, USA) using the High-Capacity cDNA Reverse Transcription Kit (4368814, ThermoFisher Scientific, Waltham, MA, USA) and Oligo(dT)18 Primer (SO131, ThermoFisher Scientific, Waltham, MA, USA). qRT-PCR was conducted in 384-well plates (ThermoFisher Scientific) with SYBR Green PCR Master Mix (4309155, Applied Biosystems, Warrington, UK) following standard conditions: 2 min at 50°C, 10 min at 95°C, 40 cycles of 15 s at 95°C, 1 min at 60°C, and 15 s at 95°C, followed by 15 s at 60°C and 15 s at 95°C in the 7900HT Fast Real-Time PCR System (ThermoFisher Scientific). Each sample was analyzed in triplicate, and the relative fold change in gene expression levels was calculated using the formula 2-ddCt, where β-actin (ActB) and β2-Microglobulin (B2m) served as the internal control. The primer pairs used for qRT-PCR are listed in (Supplementary Table S1).
